# Relationships for Women with Breast Cancer Among Extraversion and Neuroticism Personality, Stress, Demoralization, Sleep Disturbance, and Psychological Well-Being: A Structural Equation Model

**DOI:** 10.3390/cancers17030400

**Published:** 2025-01-25

**Authors:** Ming-Hsin Yeh, Ren-Hau Li

**Affiliations:** 1 Department of Surgery, Chung Shan Medical University Hospital, Taichung 40201, Taiwan; cshy1629@csmu.edu.tw; 2 Institute of Medicine, School of Medicine, Chung Shan Medical University, Taichung 40201, Taiwan; 3 Department of Psychology, Chung Shan Medical University, Taichung 40201, Taiwan; 4 Clinical Psychological Room, Chung Shan Medical University Hospital, Taichung 40201, Taiwan

**Keywords:** big five personality traits, demoralization, post-traumatic growth, psychological well-being, sleep disturbances, post-breast-cancer stress

## Abstract

Objectives: According to the personality five-factor theory (FFT), this study explored a structural equation model for women with breast cancer involving variables, including extraversion and neuroticism, post-breast-cancer stress, demoralization, sleep disturbances, and psychological well-being (PWB). Methods: A total of 351 women with breast cancer were recruited from mid-Taiwan for the cross-sectional questionnaire survey. Results: In this model, extraversion had direct effects on demoralization (−0.21), sleep disturbances (−0.16), and PWB (0.36); neuroticism had direct effects on post-breast-cancer stress (0.28), demoralization (0.12), and sleep disturbances (0.19); post-breast-cancer stress had direct effects on demoralization (0.71) and PWB (0.38); demoralization had direct effects on sleep disturbances (0.33) and PWB (−0.75). Accordingly, extraversion had a higher direct influence on demoralization than neuroticism (−0.21 vs. 0.12); however, neuroticism correlated with demoralization more than extraversion with demoralization (0.36 vs. −0.28). Neuroticism had a higher direct influence on sleep disturbances than extraversion on sleep disturbances. With regards to indirect influences, post-breast-cancer stress partially mediated the path from neuroticism to demoralization. Demoralization completely mediated the path from post-breast-cancer stress to sleep disturbances and partially mediated the path from extraversion to sleep disturbances. Moreover, demoralization and post-breast-cancer stress together completely mediated the path from neuroticism to PWB. Furthermore, a suppression effect occurred, making the influence from post-breast-cancer stress to PWB positive, which seemed to characterize post-traumatic growth; meanwhile, the suppression enforced the negative effect of demoralization on PWB. Conclusions: Post-breast-cancer stress and demoralization play important roles in clinical counseling for survivors of women with breast cancer.

## Simple Summary

There were few studies to build a path model on extraversion and neuroticism personality, post-breast-cancer stress, demoralization, sleep disturbances, and psychological well-being for survivors of women with breast cancer. Based on the personality five-factor theory (FFT), a structural equation model was built to help understand these psychosocial variables’ interplay processes. It showed that the extraversion and neuroticism personality traits had different influential paths on post-breast-cancer stress, demoralization, sleep disturbances, and psychological well-being. By describing different direct effects, indirect effects, and a suppression effect among these psychosocial variables with path coefficients, the complicated path relationships were understood and discussed. The paths make counseling with survivors of women with breast cancer more insightful. The path model implied that post-breast-cancer stress and demoralization played important roles in helping women survivors of breast cancer.

## 1. Introduction

Breast cancer remains the leading cancer diagnosis among women, which reaches 31% of all women cancers [1]. In Taiwan, it is particularly prevalent in women aged 45–69, with over 10,000 new cases annually and approximately 2000 deaths each year; however, early detection significantly enhances the five-year survival rate to over 90% [2]. Among cancer survivors, breast cancer survivors constitute 24%, leading to an increasing focus on post-treatment quality of life [3,4], indicating that psychological traits may play a crucial role in the disease’s trajectory and survivors’ quality of life [5]. More recent studies have suggested that specific personality characteristics, particularly extraversion and neuroticism, may influence emotional responses (e.g., stress responses, demoralization), sleeping disturbances, and quality of life following a breast cancer diagnosis [6]. Stress, emotional responses, sleep disturbances, and adaptation have long been important issues in studies of cancer patients. A systematic review indicated that stress and demoralization were associated with negative health outcomes [6]. As for psychological well-being, it is close to the concept of quality of life [7,8] but emphasizes more the rational and positive aspects than emotional well-being and is, therefore, suitable to be used as an assessment of long-term and ultimately positive psychological adaptation in breast cancer patients [9]. As yet, there are fewer empirical studies to construct a model connecting such related variables for breast cancer patients. The present research aimed to build a structural equation model involving personality traits (extraversion and neuroticism), psychosocial variables (post-breast-cancer stress, demoralization, and sleep disturbances), and psychological well-being.

## 2. Literature Reviews

### 2.1. Influences from Personality Traits

Personality traits, particularly extraversion and neuroticism, have been consistently found to predict well-being and emotional outcomes in cancer patients. Severe life events and depression were the strongest predictors of breast cancer, and depression was inextricably linked with neuroticism [10]. A study with 203 survivors after breast cancer surgery also found that neurotic personality could predict quality of life and emotional functioning [11]. Studies [12,13] have demonstrated that higher levels of neuroticism predict lower quality of life and higher stress, while extraversion correlates with optimism, better coping, and higher life satisfaction in breast cancer survivors.

The Five Factor Theory (FFT) [14,15] describes the dynamic processes among basic tendencies (the five personality traits), characteristic adaptations, self-concept (also a part of characteristic adaptations), objective biography, external influences, and biological bases. The basic tendencies are abstract psychological potentials, while characteristic adaptations are concrete manifestations (habits, attitudes, skills, roles, and relationships). The present research included women with the biological bases of breast cancer as the context of external influences, exploring the path relationships among their personality traits (extraversion and neuroticism), two characteristic adaptations (post-breast-cancer stress, demoralization), an objective biography (sleep disturbances), and a self-concept (psychological well-being).

### 2.2. Post-Breast-Cancer Stress and Demoralization

In the present research, post-breast-cancer stress was defined as the continuing effects of stress after having breast cancer and the fear of deterioration caused by breast cancer itself. Previous research has shown that stress about breast cancer recurrence is a significant risk factor for depression [16,17] and that recurrence survivors have worse emotional well-being, hope, and other physical and psychological functioning problems compared to non-recurrence survivors [18]. Breast cancer recurrence is often devastating news for patients [4]. Therefore, post-breast-cancer stress could be viewed as a negative characteristic adaptation.

Demoralization and depression are common and important responses to stressful situations. Demoralization usually occurs in breast cancer patients before they reach depression. Demoralization is associated with a subjective sense of incompetence, whereas depression is associated with anhedonia [19]; demoralization is a loss of hope, meaning, and anticipated joy rather than an overall inability to be joyful [20,21]. Some research also found that the demoralization of women with breast cancer was more predictive of quality of life than depression [22]. Moreover, according to the Big Five theory [14,15], demoralization could be viewed as a kind of negative adaptation characteristic after suffering breast cancer, which may influence sleep quality and negative behaviors later on. In the present research, demoralization was used instead of depression.

### 2.3. Sleep Disturbances

Sleep disturbances have also been identified as a common issue in breast cancer survivors, often exacerbated by chemotherapy, hormonal treatments, and anxiety about recurrence. Poor sleep quality has been linked to depression, fatigue, and lower psychological well-being. Sleep quality among breast cancer patients often deteriorates post-treatment, with severe implications for overall health and emotional functioning [23,24]. Sleep is arguably an important aspect of quality of life, and many physical and psychological problems related to poor sleep quality occur after cancer [23,25]. Recent medical research has also found that breast cancer tumor cell metastasis occurs during the sleep–rest phase [26], suggesting that sleep quality is related to the quality of life of breast cancer patients. The prevalence of sleep problems among breast cancer survivors is as high as 39% [27], with those with severe sleep problems having significantly poorer role functioning, physical pain, role mood, and psychological well-being than those with good sleep quality [28]. In a comparison between neuroticism and critical personality, the former had a significant effect on subjective sleep disturbance, while the latter was significantly associated with objective sleep indicators [29]. Thus, it is likely that neuroticism plays an important role in the assessment of subjective sleep disturbances and the resulting depression [30]. In the present research, sleep quality was measured by the Pittsburgh Sleep Quality Index (PSQI) [31], which includes some objective indices and, therefore, could be viewed as an objective biography, not only a subjective report.

### 2.4. Psychological Well-Being

Psychological well-being is a kind of more eudaimonic well-being than subjective emotional well-being [32,33]. Breast cancer patients typically have higher levels of anxiety and depression and lower levels of physical and mental health [34], and the use of psychological well-being, which focuses on the eudaimonic spirituality that positive psychology emphasizes, to observe patient encounters from a positive perspective, may extend the findings of studies related to quality of life of breast cancer patients [11,12], as well as expanding the findings on emotional well-being and interpersonal well-being of breast cancer patients [35]. Past research on well-being has found an important relationship with personality traits [36,37,38]. Some studies found that neuroticism and extraversion are the two strongest explanations of subjective well-being among the Big Five traits of personality [8,39].

### 2.5. The Model of Relationships Among Personality Traits, Post-Breast-Cancer Stress, Demoralization, Sleep Disturbance, and Psychological Well-Being

**Figure 1:**
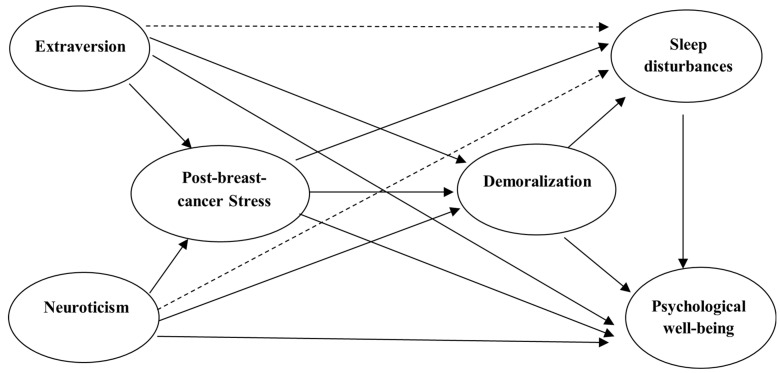
A path relationship theory model for extraversion, neuroticism, post-breast-cancer stress, demoralization, sleep disturbances, and psychological well-being. Note: Paths with solid lines were based on FFT, while dashed lines were based on the literature.

The FFT [14,15] posits that personality traits remain relatively stable across an individual’s lifespan but significantly influence how one responds to life-altering events like a cancer diagnosis. A structural model based on FFT is shown in Figure 1. The complete structural equation model with measurement models is presented in Figure S1 in the Supplementary Materials. Starting with the five-factor theory of personality in the context of those extrinsic influences related to breast cancer. Extraversion and neuroticism personality traits are considered to connect with their effects on post-breast-cancer stress and demoralization (two kinds of characteristic adaptations), sleep disturbances (sleep behavior, a kind of objective biography), and psychological well-being (a kind of self-concept, a cognitive self-appraisal that is a rather stable appraisal of one’s own life). According to FFT, external influences (such as events related to breast cancer) influence biological bases, characteristic adaptations, and objective biography. Biological bases then influence basic tendencies (personality traits). Personality traits influence characteristic adaptations and self-concepts. The characteristic adaptations then influence objective biography and self-concept. The objective biography also influences self-concept. The paths with solid lines mainly followed the FFT, and the two paths with dashed lines were complementary, according to the findings from the literature.

## 3. Materials and Methods

### 3.1. Sample

This study was approved by the Institute Review Board of Chung-Shan Medical University (CSMUH No: CS-13203 and CS1-20158). Women with breast cancer were first asked about their willingness to participate in the survey and signed a consent form before completing the questionnaire. The participants were selected based on the following criteria: (1) intention to undergo active treatment (including surgery, chemotherapy, or radiotherapy; at least one of the three was completed); (2) over 20 years of age and under 80 years of age; (3) not suffering from serious mental illnesses, such as schizophrenia, major depression, etc. This study’s data were mainly collected in the Taichung area of Taiwan, and a total of 351 valid samples were obtained.

### 3.2. Instruments

#### 3.2.1. BFI-15 Big Five Personality Inventory

A 15-item simplified version of the Big Five Inventory [BFI] [40] came from its original 44-item [41] version, which is scored on a five-point Likert scale ranging from very imprecise 1 point to very precise 5 points. The convergent validity was established with another Big Five Personality Scale (TDA). The internal consistency alpha coefficients of the BFI-15 ranged from 0.67 to 0.81, and factor loadings ranged from 0.44 to 0.87. In the present study, only two subscales, Extraversion and Neuroticism Personality, were used, with three items each. The two subscales had internal consistency alpha coefficients 0.76 and 0.74, respectively, and had factor loadings ranging from 0.58 to 0.83.

#### 3.2.2. Psychological Well-Being Scale

The 18-item Psychological Well-Being Scale [42,43] was used, which is a reduced version of its 84-item version [44]. It had six factors, including self-acceptance, positive relationships with others, autonomy, environmental mastery, purpose of life, and personal growth, and scored on a six-point Likert scale ranging from strongly disagree, with 1 point, to strongly agree, with 6 points. The higher scores represent the higher psychological well-being. The 18-item version had excellent confirmatory factor analysis results, with internal consistency alpha coefficients ranging from 0.62 to 0.76 for each subscale and total scale alpha reliabilities as high as 0.93. The present sample showed internal consistency alpha coefficients ranging from 0.83 to 0.91; the reliability of the total scale was 0.93, and the factor loadings ranged from 0.73 to 0.93.

#### 3.2.3. The Stress of Breast Cancer After Primary Therapy Scale

The stress of breast cancer after primary therapy scale was adopted [45] to measure post-breast-cancer stress, which included three subscales: feelings of unpredictability; feelings of uncontrollability; and feelings of psychological burden, which had good reliability and construct validity. The scale was based on a four-point Likert scale, with 0 representing “completely disagree” and 3 representing “completely agree”, with higher scores representing greater stress. The present sample showed an internal consistency alpha coefficient ranging from 0.68 to 0.79, total scale reliability of 0.89, and factor loadings ranging from 0.50 to 0.84.

#### 3.2.4. Pittsburgh Sleep Quality Inventory

The Pittsburgh Sleep Quality Index (PSQI) [31] was used to measure the sleep disturbances of women with breast cancer in the past month. There were 19 questions, and seven components had to be calculated in a specific way, which were subjective sleep quality, sleep latency, sleep duration, habitual sleep efficiency, sleep disturbance, use of sleeping medication, and daytime dysfunction. The score for each component ranges from 0 to 3, with higher scores indicating more serious sleep impairment and dysfunction. The internal consistency coefficient alpha of the seven components was 0.83, and the test–retest reliability of the total score at about one-month intervals was 0.85. In terms of construct validity, the total score can significantly differentiate between sleep disorders and the control group [46]. The present sample showed an internal consistency coefficient alpha of 0.75, with factor loadings ranging from 0.38 to 0.85.

#### 3.2.5. Demoralization Scale

The Demoralization Scale [47], translated from its English version [48], was used, which consists of 24 items divided into 5 subscales, including loss of meaning and purpose, dysphoria, disheartenment, helplessness, and sense of failure. Each item used Likert’s five-point scoring, and higher scores represented higher demoralization. The scale had internal consistency coefficients alpha ranging from 0.63 to 0.88 for each subscale and 0.92 for the total scale. The present sample showed an internal consistency alpha coefficient ranging from 0.81 to 0.90, a total scale reliability of 0.96, and factor loadings ranging from 0.63 to 0.91.

### 3.3. Statistical Analysis

Raw data have less than 7% missing values for background variables and less than 3% missing values for continuous variables. The multiple imputation method was then used to find the missing values of continuous variables. Statistical software SPSS version 23 [49] was used for screening data and descriptive statistics. Moreover, LISREL version 8.8 [50] was used to construct a structural equation model with the maximum likelihood estimation method for relationships among extraversion, neuroticism, post-breast-cancer stress, demoralization, sleep disturbances, and psychological well-being. Model-fit indices such as chi-square statistic (χ^2^), degrees of freedom (*df*), *p*-value, χ^2^/*df*, root mean square error of approximation (RMSEA), standardized root mean square residual (SRMR), comparative fit index (CFI), goodness-of-fit index (GFI), and non-normed fitness index (NNFI) were reported. Specifically, *p*-value > 0.05, χ^2^/*df* < 3, CFI > 0.95, GFI > 0.95, NNFI > 0.95, SRMR < 0.08, and RMSEA < 0.07 are preferred. For the number of parameters estimated (sixty-nine) in the model without modification, five times the number is 69 × 5 = 345; at least 345 participants were needed. Our study sample was 351 participants, which met the standard.

## 4. Results

### 4.1. Descriptive Statistics and Latent Variables Correlation

**Figure 2:**
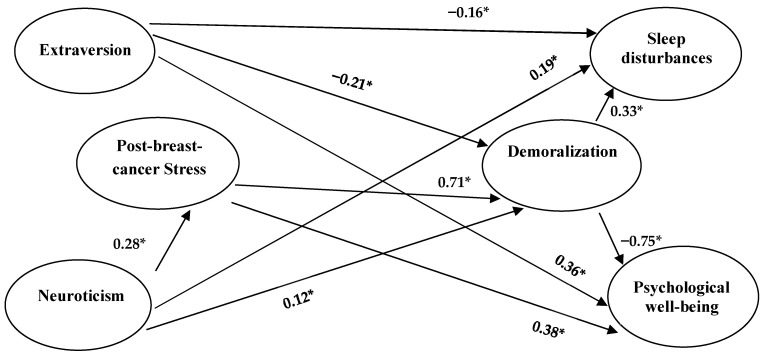
Completely standardized parameter estimates for the structure paths of the structural model. Note: * All the paths in the figure were significant at least 0.01 level.

The demographic information of 351 women with breast cancer is shown in Table 1. The mean age was 51.82 years, with a standard deviation of 8.56 years, ranging from 29 to 76 years. The women had been suffering from breast cancer for 0.87 to 1237 months, with a mean of 38.6 months and a median of 29 months. Regarding the stage of breast cancer, 146 women (41.6%) had stage 2 cancer, and 112 women (31.9%) had stage 1 cancer. In addition, in terms of counts, 87.5% received surgery; 64.4% received radiation therapy; 67.5% received chemotherapy, and 41.0% received hormone therapy. Moreover, 268 (76.4%) women were married, and 199 (56.7%) women had an educational level of senior high school/vocational school. The average monthly income was less than USD 20,000 for 158 (45.0%) women and USD 20,000 to USD 50,000 for 130 (37.0%) women. As for menopausal status, 293 (83.5%) women had stopped menstruating. The latent variable correlation coefficients in Table 2 could be used against the structural path coefficients in Figure 2 to help understand the model. Moreover, the descriptive statistics and simple correlation coefficients between observed continuous variables were presented in Table S1 as Supplementary Materials.

According to the model in Figure S1 and the sample data collected, model-fit outcomes show that the χ^2^ is 912.36, *df* = 309, *p* < 0.001, the RMSEA = 0.075, the SRMR = 0.064, the CFI = 0.95, the NNFI = 0.94, and the GFI = 0.84, which indicated that the model matched the data roughly. To obtain more accurate results with better model fit, the model could be modified by setting the correlation of the residuals of the observed variables [45]. In this study, we used the chi-square critical value of 3.84 for one df at the 0.05 significance level as a criterion and set the correlation of the errors above this value according to the modification index except for two error correlations to avoid Heywood cases. In addition, four insignificant path structure coefficients were also removed from this model, as shown in Figure 2. After this adjustment, the chi-square value of model-fit was 342.84, the *df* was 267, *p* = 0.001, RMSEA was 0.028, SRMR was 0.051, CFI was 0.99, NNFI was 0.99, and GFI was 0.93, which showed that most of the indices were acceptable. Moreover, this model was presented with only its structural equation model in Figure 2 for simplicity, and the estimated coefficients in the measurement models are listed in Table S2 as Supplementary Materials. The total effects and indirect effects are presented in Table 3.

Figure 2 shows that the proportion of variance accounted for was 8% for post-breast-cancer stress, 64% for demoralization, 26% for sleep disturbances, and 54% for psychological well-being. The effects of the other three constructs were analyzed in detail below.

### 4.2. Psychological Well-Being

Figure 2 shows that the proportion of variance accounted for psychological well-being was as high as 54%. Extraversion, post-breast-cancer stress, and demoralization had a direct effect on psychological well-being (0.36, 0.38, −0.75, respectively), in which two of the paths appeared to have implausible coefficients. That is, demoralization, pointing to psychological well-being, had a coefficient as high as −0.75, which exceeded the simple correlation coefficient of −0.57. Post-breast-cancer stress pointing to psychological well-being had a positive coefficient of 0.38, which had the opposite sign to the negative correlation coefficient of −0.21. Taken together, the phenomenon of suppression effect occurred [51]. In this three-variable relationship, the post-breast-cancer stress suppressed certain components of psychological well-being that would interfere with the interpretation of psychological well-being by demoralization and, thus, contribute to the greater-than-expected effect of demoralization on psychological well-being.

Moreover, compared with Figure 1, the effect of sleep disturbances on psychological well-being disappeared due to the competing effects of extraversion, post-breast-cancer stress, and demoralization. Further testing (in private) revealed that sleep disturbances had a significant direct effect on psychological well-being only when the path coefficient of demoralization on psychological well-being was taken away. Coupled with the fact that the direct effect of demoralization on psychological well-being was as high as −0.75, it could be seen that the effect of demoralization on psychological well-being was very important.

From the perspective of mediating effects, the effects of neuroticism on psychological well-being could be mediated by post-breast-cancer stress and demoralization with three mediating paths, two of which were simple mediation of post-breast-cancer stress or demoralization, with two mediating effects of 0.28 × 0.38 = 0.11 (*p* < 0.001 in a Sobel test) and 0.12 × (−0.75) = −0.09 (*p* = 0.007), and the other was the multiple mediation of post-cancer stress and demoralization, with a mediating effect of 0.28 × 0.71 × (−0.75) = −0.15 (*p* < 0.001). Meanwhile, the relationship between neuroticism and psychological well-being changed from a significant coefficient (−0.12, *p* = 0.027, controlling for extraversion) to an insignificant path coefficient, which could be considered completely mediated. In addition, if the path from extraversion to psychological well-being had been taken away, the path coefficient of neuroticism on psychological well-being would still be insignificant, and a completely mediated phenomenon would still exist. In contrast, extraversion still had a direct positive effect (0.36) on psychological well-being while there existed a mediating effect through demoralization (i.e., (−0.21) × (−0.75) = 0.16, *p* < 0.001, refer to Table 3). It also meant that the extraversion’s positive total effects (0.52, *p* < 0.001) could mitigate the negative total effects (−0.13, *p* < 0.001) from neuroticism on psychological well-being (refer to Table 3).

### 4.3. Sleep Disturbances

The proportion of variance in sleep disturbances explained by extraversion, neuroticism, and demoralization was 24%, which was a large effect size (above 14%) [52]. Demoralization had a relatively large direct effect of 0.33. Neuroticism had slightly more of a direct effect than extraversion (0.19 and −0.16, respectively). In terms of mediating effects, extraversion had a partial mediating effect of −0.07 (−0.21 × 0.33, *p* < 0.001, refer to Table 3) on sleep disturbances through demoralization. In contrast, neuroticism had a partial mediating effect of 0.04 (0.12 × 0.33, *p* = 0.011) on sleep disturbances through demoralization and had a multiple mediating effect of 0.07 (0.28 × 0.71 × 0.33, *p* < 0.001) on sleep disturbances through post-breast-cancer stress and demoralization. In comparison, the indirect effects of neuroticism on sleep disturbances remained higher than that of extraversion (refer to Table 3). In addition, the relationship between post-breast-cancer stress and sleep disturbances changed from a significant path coefficient (0.19 in private testing, *p* = 0.001, when controlling for extraversion and neuroticism) to an insignificant path coefficient, with a complete mediating effect of 0.23 (0.71 × 0.33, *p* < 0.001, refer to Table 3) through demoralization. It was worth noting that also, when the path of neuroticism’s direct effect on sleep disturbances was removed and the direct effect of extraversion on sleep disturbances was retained, this complete mediation still held, which showed that the ability of demoralization to mediate between the two was rather large, and also emphasized that neuroticism had the power to influence sleep disturbances independently of post-breast-cancer stress. Although neuroticism was a stable personality trait that was difficult to change in a short time, if demoralization was properly addressed, the negative effects of post-breast-cancer stress on sleep quality could be greatly reduced when demoralization was carefully dealt with. Therefore, demoralization was a variable that played a very important role in the post-treatment rehabilitation of women with breast cancer.

### 4.4. Demoralization

As much as 64% of the variance in demoralization was explained by extraversion, neuroticism, and post-cancer stress, with a direct effect of 0.71 from post-breast-cancer stress being the main key. Furthermore, extraversion had no mediating effect on demoralization, whereas neuroticism’s effect on demoralization was partially mediated by post-breast-cancer stress, with a mediating effect of 0.20 (0.28 × 0.71, *p* < 0.001, refer to Table 3). It is noteworthy that extraversion had a greater direct effect on demoralization (−0.21) than neuroticism (0.12); however, the simple correlation between demoralization and neuroticism (*r* = 0.36) was higher than the simple correlation with extraversion (*r* = −0.28), which suggested that post-breast-cancer stress played an important mediating role between neuroticism and demoralization. Dealing with post-breast-cancer stress would decrease the negative effect of demoralization from neuroticism.

## 5. Discussion

The post-breast-cancer stress as a negative adaption characteristic in the immediate aftermath of a major traumatic event (breast cancer) was found to have a specific effect in the present study. In terms of direct effects, post-breast-cancer stress had a surprisingly positive effect on psychological well-being as an indicator of psychological adjustment. However, in terms of indirect effects, post-cancer stress had a conceptually plausible negative effect on psychological well-being through demoralization; when comparing the two effects, the negative indirect effect was much stronger than the positive direct effect, and, therefore, the overall effect of post-breast-cancer stress was still detrimental to psychological well-being, which was consistent with the simple negative correlation between the two constructs and the findings from a similar model in the past [45]. It was worth mentioning the existence of a positive direct effect on psychological well-being in patients who had experienced the trauma of cancer. This may characterize the psychological growth that occurs when patients experience cancer trauma [9,53,54] and was consistent with post-traumatic growth (PTG) theory [55], which was associated with personality traits, such as optimism or positive affective traits [56]. In the present study, post-traumatic growth existed when considering extraversion and neuroticism personalities in the model, suggesting that other factors not covered in the present study still had a role to play [57].

While past research had indicated that extraversion had a high positive correlation with psychological well-being and neuroticism had a moderate negative correlation with psychological well-being [58], the present study also found the same results using a sample of breast cancer patients. It meant that breast cancer patients, like the general population, did not specifically highlight that neuroticism personality had a greater impact on psychological well-being than extraversion personality. In addition, there still existed a direct effect of extraversion on psychological well-being besides the mediation effect through demoralization, but the direct effect of neuroticism on psychological well-being was completely mediated by post-breast-cancer stress and demoralization. This indicated that extraversion still had a more direct effect on psychological well-being than neuroticism in breast cancer patients.

The present study was consistent with past research in that neuroticism had a higher simple correlation with the two negative adaption characteristics, post-breast-cancer stress, and demoralization, than extraversion [59,60]. Moreover, the present study found that post-breast-cancer stress mediated most of the neuroticism’s effect on demoralization, whereas extraversion’s effect on demoralization was more direct. Furthermore, the present study found that neuroticism was more related to sleep disturbance than extraversion, which was also consistent with past studies [31,61]. Finally, the present study also found that the direct effect of neuroticism on sleep disturbance was higher than extraversion, but extraversion had a higher indirect effect through demoralization on sleep disturbances than neuroticism. However, if the multiple indirect (mediating) effect from neuroticism through post-breast-cancer stress and then demoralization to sleep disturbances was considered, neuroticism still had higher indirect effects on sleep disturbances than extraversion. It was worth noting that this significant multiple-mediating effect characterized the influence power from neuroticism to a distant outcome variable. It was consistent with some research emphasizing the severe negative outcomes related to neuroticism [10,11,12].

From a neuroplasticity perspective, damage to organs has been shown to produce corresponding changes in the corresponding cortical areas of the brain [62], and whether or not the effects caused by breast cancer on the body also produce corresponding effects in the brain remains to be investigated by more focused empirical studies. The development of breast cancer might be associated with certain genetic mutations and their inheritance, and patients themselves might have a familial genetic predisposition to breast cancer [63]. Other non-genetic risk factors also affected breast carcinogenesis [64,65]. The development of breast cancer was a traumatic event caused by a relevant extrinsic influence [64], and these extrinsic influences might also directly affect adaption-related traits, not necessarily indirectly through personality traits. According to the FFT, these peripheral extrinsic elements were not usually the focus of personality psychology theories. Thus, whichever pathway the external influences associated with breast cancer took to affect other constructs was not the focus of the theory [16] and of the present research.

The limitations of the present research were as follows. Firstly, only the cross-sectional questionnaire survey was used, making the causal path model less credible. A longitudinal study design could be used to confirm these research results. Secondly, background variables were not considered control variables in the model because of missing values and insufficient sample size to guarantee the quality of parameter estimates in a more complicated model. A larger sample size will be needed to test its related derived models. Finally, even if the fit indices of the model are acceptable, it could not be over-interpreted as the model is correct. It only means that the model was consistent with the data. It is always possible that a different model could fit the data equally well.

## 6. Clinical Implications

The findings of this study had important implications for clinical practice. Given the significant role that personality traits play in shaping psychological outcomes, healthcare providers should consider personality assessments as part of routine care for breast cancer patients. Tailoring interventions based on personality traits could enhance the effectiveness of psychological support and improve overall well-being. For example, patients high in neuroticism might benefit from more intensive stress management and cognitive–behavioral interventions, while those high in extraversion might respond well to group therapy or interventions that encourage social support and engagement. Additionally, addressing demoralization should be a priority in the care of breast cancer patients, as this factor significantly impacts sleep disturbances and psychological well-being. When demoralization is considered and dealt with adequately in clinical practices, sleep quality would be improved, and psychological well-being would undertake less harm. Furthermore, adequately guiding post-breast-cancer stress to the growth of mind and spirit would positively promote psychological well-being to obtain post-traumatic growth experiences. Multidisciplinary care teams, including oncologists, psychologists, and sleep specialists, should work together to provide comprehensive care that addresses both the physical and psychological needs of breast cancer survivors.

## 7. Conclusions

This study reinforced the importance of considering psychological factors, particularly personality traits, in the treatment and care of breast cancer patients. This study highlighted key research findings in these areas, mainly focusing on neuroticism and extraversion and the interplay of these traits with various psychological outcomes in breast cancer survivors. Specifically, extraversion could directly influence demoralization, sleep disturbances, and psychological well-being, in which demoralization also served as a mediator. Meanwhile, neuroticism could directly influence post-breast-cancer stress, demoralization, and sleep disturbances and indirectly influence psychological well-being, in which post-breast-cancer stress and demoralization also served as mediators. Healthcare providers should consider a multidisciplinary approach that includes oncologists, psychologists, and sleep specialists to ensure comprehensive care involving post-breast-cancer stress, demoralization, and sleep disturbances that address both the physical and emotional needs of women with breast cancer. By understanding the complex process mechanisms between personality traits, psychosocial factors (post-breast-cancer stress, demoralization, and sleep disturbances), and psychological well-being, healthcare teams could develop more personalized treatment plans accordingly that supported both the mental and physical recovery of women with cancer.

## Figures and Tables

**Table 1:** Demographic information of women with breast cancer (N = 351).

Background Variables	Number	Percentage (%)
Number of months with cancer		
Within 12 months	68	19.4
12–36 months	137	39.0
36–60 months	94	26.8
Over 60 months	44	12.5
Missing	8	2.3
Diagnostic Stages of Breast Cancer		
Stage 0	21	6.0
Stage 1	112	31.9
Stage 2	146	41.6
Stage 3	53	15.1
Stage 4	10	2.8
Missing	9	2.6
Treatment type		
Surgery	307	87.5
Radiation therapy	226	64.4
Chemotherapy	237	67.5
Hormone Therapy	144	41.0
Missing	24	6.8
Marital status		
Unmarried	33	9.4
Married	268	76.4
Separated	31	8.8
Widowed	14	4.0
Missing	5	1.4
Educational level		
Illiterate	5	1.4
Elementary/junior high school	69	19.7
Senior high school/vocational school	199	56.7
University and above	73	20.8
Missing	5	1.4
Monthly income (NWD)		
Below 20,000	158	45.0
20,000–50,000	130	37.0
50,000–80,000	36	10.3
Over 80,000	17	4.8
Missing	10	2.8
Menopause Status		
Stopped menstruating	293	83.5
Not Stopped	49	14.0
Missing	9	2.6

Note: In the treatment type, some women received at least one kind of treatment type.

**Table 2:** Simple correlation coefficients between the six latent variables (N = 351).

	1	2	3	4	5	6
1. Extraversion	1.00					
2. Neuroticism	−0.23 ***	1.00				
3. Post-cancer stress	−0.06	0.28 ***	1.00			
4. Demoralization	−0.28 ***	0.36 ***	0.75 ***	1.00		
5. Sleep disturbances	−0.29 ***	0.35 ***	0.26 ***	0.45 ***	1.00	
6. Psychol. well-being	0.55 ***	−0.26 ***	−0.21 ***	−0.57 ***	−0.36 ***	1.00

Note: *** *p* < 0.001.

**Table 3:** Total effects and indirect effects of the structural model (N = 351).

Total Effects (Direct Effect Plus Indirect Effects)	Standardized Estimates	S.E.	t
Extraversion → Sleep disturbances	−0.23 ***	0.061	−3.80
Extraversion → Demoralization	−0.21 ***	0.042	−4.99
Extraversion → Psychological well-being	0.52 ***	0.068	7.60
Neuroticism → Sleep disturbances	0.30 ***	0.061	4.83
Neuroticism → Post-breast-cancer stress	0.28 ***	0.058	4.76
Neuroticism → Demoralization	0.31 ***	0.060	5.18
Neuroticism → Psychological well-being	−0.13 ***	0.035	−3.69
Post-breast-cancer stress → Sleep disturbances	0.23 ***	0.045	5.20
Post-breast-cancer stress → Demoralization	0.71 ***	0.049	14.43
Post-breast-cancer stress → Psychological well-being	−0.15 **	0.052	−2.95
Demoralization → Sleep disturbances	0.33 ***	0.061	5.41
Demoralization → Psychological well-being	−0.75 ***	0.094	−7.98
**Indirect effects (Mediating effects)**			
Extraversion → Sleep disturbances	−0.07 ***	0.019	−3.72
Extraversion → Psychological well-being	0.16 ***	0.036	4.43
Neuroticism → Sleep disturbances	0.10 ***	0.026	4.01
Neuroticism → Demoralization	0.20 ***	0.042	4.64
Neuroticism → Psychological well-being	−0.13 ***	0.035	−3.69
Post-breast-cancer stress → Sleep disturbances	0.23 ***	0.045	5.20
Post-breast-cancer stress → Psychological well-being	−0.53 ***	0.072	−7.41

Note: ** *p* < 0.01; *** *p* < 0.001.

## Data Availability

The data presented in this study are available upon request, owing to privacy restrictions.

## Supplementary Materials

The following supporting information can be downloaded at https://www.mdpi.com/article/10.3390/cancers17030400/s1, Figure S1. Complete structural equation model based on FFT and the literature; Table S1: Descriptive statistics and simple correlation coefficients between observed variables; Table S2: Estimated coefficients in the measurement models.
