# Supplementary material for: Relationships for Women with Breast Cancer Among Extraversion and Neuroticism Personality, Stress, Demoralization, Sleep Disturbance, and Psychological Well-Being: A Structural Equation Model
Source: Cancers (Basel). 2025 Jan 25;17(3):400. doi: 10.3390/cancers17030400 (PMC11816334; doi:10.3390/cancers17030400)
Supplement: Supplementary file 1 [file cancers-17-00400-s001.zip › cancers-3402030-supplementary.pdf]

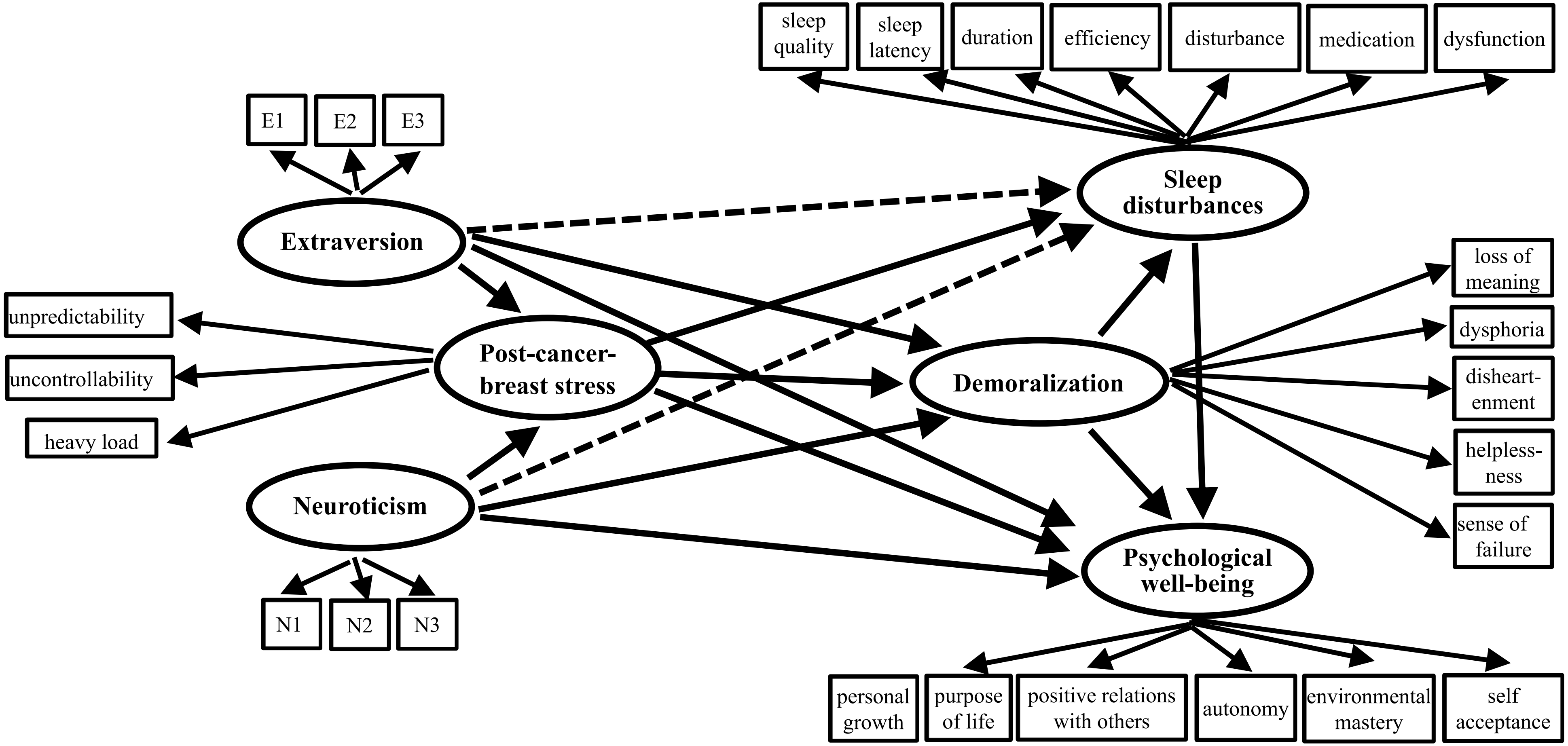

Figure S1. Complete structural equation model based on FFT and literature

Table S1. Descriptive statistics and simple correlation coefficients between observed variables (N = 351)

	E1	E2	E3	N1	N2	N3	St1	St2	St3	D1	D2	D3	D4	D5	Sl1	Sl2	Sl3	Sl4	Sl5	Sl6	Sl7	P1	P2	P3	P4	P5	P6
E1	1.0																										
E2	.64*	1.0																									
E3	.48*	.46*	1.0																								
N1	-.11*	-.04	-.13*	1.0																							
N2	-.26*	-.18*	-.09	.47*	1.0																						
N3	-.21*	-.12*	-.19*	.54*	.45*	1.0																					
St1	.01	-.08	.06	.09	.28*	.06	1.0																				
St2	-.03	-.09	.03	.13*	.31*	.09	.87*	1.0																			
St3	-.03	-.10	.00	.14*	.36*	.14*	.87*	.85*	1.0																		
D1	-.16*	-.22*	-.05	.06	.36*	.06	.58*	.59*	.61*	1.0																	
D2	-.21*	-.23*	-.14*	.28*	.49*	.31*	.58*	.58*	.64*	.68*	1.0																
D3	-.20*	-.23*	-.11*	.20*	.44*	.17*	.63*	.64*	.68*	.76*	.84*	1.0															
D4	-.17*	-.18*	-.06	.13*	.42*	.19*	.54*	.59*	.56*	.78*	.75*	.77*	1.0														

D5	-.18*	-.26*	-.09	.01	.26*	.01	.54*	.53*	.56*	.70*	.54*	.67*	.59*	1.0													
Sl1	-.27*	-.16*	-.14*	.24*	.35*	.20*	.15*	.25*	.22*	.31*	.35*	.35*	.32*	.26*	1.0												
Sl2	-.21*	-.13*	-.10	.14*	.24*	.18*	.06	.12*	.14*	.25*	.25*	.24*	.28*	.20*	.56*	1.0											
Sl3	-.07	-.05	-.03	.14*	.14*	.05	.05	.08	.11*	.15*	.12*	.12*	.15*	.14*	.37*	.24*	1.0										
Sl4	-.13*	-.06	-.04	.01	.16*	.04	.08	.09	.12*	.19*	.17*	.17*	.19*	.21*	.40*	.39*	.54*	1.0									
Sl5	-.15*	-.12*	-.14*	.18*	.24*	.18*	.11*	.20*	.20*	.23*	.32*	.28*	.24*	.14*	.44*	.36*	.17*	.23*	1.0								
Sl6	-.11*	-.06	.01	.06	.20*	.03	.20*	.27*	.22*	.26*	.22*	.28*	.25*	.24*	.51*	.38*	.22*	.21*	.27*	1.0							
Sl7	-.15*	-.03	-.15*	.23*	.25*	.25*	.10	.17*	.13*	.17*	.33*	.30*	.26*	.13*	.38*	.29*	.07	.08	.33*	.23*	1.0						
P1	.30*	.39*	.34*	-.01	-.14*	-.06	-.12*	-.12*	-.12*	-.30*	-.25*	-.33*	-.26*	-.36*	-.17*	-.19*	-.14*	-.17*	-.21*	-.01	-.13*	1.0					
P2	.20*	.25*	.17*	-.10	-.17*	-.10	-.06	-.06	-.08	-.20*	-.22*	-.25*	-.21*	-.32*	-.20*	-.17*	-.05	-.13*	-.15*	-.06	-.09	.43*	1.0				
P3	.24*	.25*	.12*	-.10	-.25*	-.13*	-.11*	-.12*	-.17*	-.39*	-.35*	-.33*	-.38*	-.36*	-.17*	-.18*	-.05	-.10	-.17*	-.04	-.18*	.46*	.53*	1.0			
P4	.30*	.37*	.22*	-.04	-.20*	-.02	.00	-.06	-.05	-.32*	-.25*	-.29*	-.30*	-.34*	-.17*	-.18*	-.11*	-.16*	-.19*	.03	-.05	.56*	.43*	.49*	1.0		
P5	.27*	.31*	.17*	-.09	-.18*	-.03	-.09	-.12*	-.10	-.41*	-.27*	-.31*	-.34*	-.39*	-.20*	-.18*	-.11*	-.11*	-.18*	-.08	-.08	.46*	.40*	.47*	.69*	1.0	
P6	.38*	.40*	.27*	-.22*	-.28*	-.18*	-.14*	-.19*	-.21*	-.40*	-.44*	-.47*	-.42*	-46*	-.26*	-.27*	-.16*	-.18*	-.25*	-.09	-.24*	.48*	.45*	.53*	.52*	.57*	1.0
Mean	3.48	3.45	3.32	3.62	2.56	3.45	6.54	5.88	7.01	5.16	7.87	8.37	4.88	5.64	1.56	1.68	1.20	0.81	1.65	0.78	0.92	14.19	12.80	14.70	14.90	13.66	13.40

SD	0.91	0.93	1.08	1.11	1.11	1.15	5.51	4.66	6.01	3.53	4.33	5.09	3.24	2.92	0.86	0.84	0.94	1.04	0.63	1.19	0.80	2.51	2.80	2.48	2.62	2.93	2.75

Note: Using Pearson correlation coefficients for a total of the 27 observed variables. E1–E3 represent three extraversion item scores; N1–N3 represent three neuroticism item scores; St1–St3 indicate subscale scores, respectively, as perceptions of unpredictability, perceptions of uncontrollability, and perceptions of a heavy psychological load in the Stress of Breast Cancer after Primary Therapy Scale; D1–D5 indicate subscale scores, respectively, as loss of meaning and purpose, dysphoria, disheartenment, helplessness, and sense of failure in the Demoralization Scale; Sl1–Sl7 indicate subscale scores, respectively, as subjective sleep quality, sleep latency, sleep duration, habitual sleep efficiency, sleep disturbances, use of sleeping medication, and daytime dysfunction in Pittsburgh Sleep Quality Inventory; and P1–P6 indicate subscale scores, respectively, as Ryff's personal growth, purpose of life, positive relationships with others, autonomy, environmental mastery, and self-acceptance in the Psychological Well-Being Scale–Short Form. * represents at least $p < .05$ (two-tailed)

Table S2. Estimated coefficients in the measurement model (N = 351)

**Observed variables**	**Factor loadings**	**S.E.**	**t**	**Completely standardized factor loadings**	**Measurement errors**	**S.E.**	**t**	**Completely Standardized measurement errors**
Extraversion								
E1	0.75	0.046	16.27	0.82	0.26	0.041	6.48	0.32
E2	0.73	0.048	15.32	0.78	0.35	0.043	8.02	0.39
E3	0.63	0.058	11.02	0.59	0.77	0.066	11.67	0.66
Neuroticism								
N1	0.93	0.105	8.84	0.84	0.36	0.177	2.06	0.30
N2	1.09	0.111	9.88	0.98	0.05	0.223	0.24	0.04
N3	0.60	0.077	7.76	0.52	0.95	0.096	9.97	0.73
Post-breast-cancer stress								
St1	4.98	---	---	0.90	5.64	0.724	7.79	0.19
St2	4.15	0.130	32.04	0.88	4.80	0.552	8.70	0.22
St3	5.74	0.212	27.01	0.96	2.89	0.79	3.66	0.08
Demoralization								
D1	2.93	---	---	0.83	3.86	0.353	10.95	0.31
D2	3.86	0.195	19.81	0.90	3.60	0.383	9.39	0.19
D3	4.73	0.208	22.73	0.93	3.60	0.424	8.50	0.14

D4	2.65	0.119	22.19	0.82	3.32	0.278	11.93	0.32
D5	2.16	0.117	18.45	0.74	3.93	0.326	12.07	0.46
Sleep disturbances								
Sl1	0.75	---	---	0.88	0.17	0.034	4.99	0.23
Sl2	0.54	0.047	11.42	0.64	0.42	0.037	11.36	0.59
Sl3	0.38	0.053	7.06	0.40	0.74	0.058	12.78	0.84
Sl4	0.46	0.060	7.69	0.44	0.88	0.069	12.78	0.81
Sl5	0.31	0.035	8.79	0.50	0.29	0.024	12.40	0.75
Sl6	0.67	0.066	10.08	0.56	0.97	0.080	12.02	0.69
Sl7	0.31	0.045	7.04	0.39	0.53	0.042	12.82	0.84
Psychological well-being								
P1	1.67	---	---	0.67	3.40	0.334	10.19	0.55
P2	1.52	0.174	8.74	0.55	5.45	0.439	12.42	0.70
P3	1.61	0.160	10.03	0.65	3.57	0.304	11.74	0.58
P4	1.79	0.158	11.30	0.69	3.53	0.363	9.73	0.52
P5	2.01	0.192	10.45	0.68	4.60	0.401	11.46	0.53
P6	2.34	0.200	11.70	0.84	2.21	0.330	6.71	0.29

Note: S.E. means standard error. Maximum likelihood estimates based on the final model with modification. However, the correlation coefficient estimates between errors were not listed here for simplicity. All the factor loadings are at 0.001 significant level. All measurement error estimates and their correlation estimates (not shown) are at least 0.05 significant level except for one (N2).
